# Extracting phylogenetic signal and accounting for bias in whole-genome data sets supports the Ctenophora as sister to remaining Metazoa

**DOI:** 10.1186/s12864-015-2146-4

**Published:** 2015-11-23

**Authors:** Marek L. Borowiec, Ernest K. Lee, Joanna C. Chiu, David C. Plachetzki

**Affiliations:** Department of Entomology and Nematology, University of California, Davis, USA; Sackler Institute for Comparative Genomics, American Museum of Natural History, New York, USA; Department of Molecular, Cellular, and Biomedical Sciences, University of New Hampshire, Durham, USA

**Keywords:** Metazoa, Ctenophora, Phylogenetic conflict, Phylogenomics, Locus selection, Long-branch attraction

## Abstract

**Background:**

Understanding the phylogenetic relationships among major lineages of multicellular animals (the Metazoa) is a prerequisite for studying the evolution of complex traits such as nervous systems, muscle tissue, or sensory organs. Transcriptome-based phylogenies have dramatically improved our understanding of metazoan relationships in recent years, although several important questions remain. The branching order near the base of the tree, in particular the placement of the poriferan (sponges, phylum Porifera) and ctenophore (comb jellies, phylum Ctenophora) lineages is one outstanding issue. Recent analyses have suggested that the comb jellies are sister to all remaining metazoan phyla including sponges. This finding is surprising because it suggests that neurons and other complex traits, present in ctenophores and eumetazoans but absent in sponges or placozoans, either evolved twice in Metazoa or were independently, secondarily lost in the lineages leading to sponges and placozoans.

**Results:**

To address the question of basal metazoan relationships we assembled a novel dataset comprised of 1080 orthologous loci derived from 36 publicly available genomes representing major lineages of animals. From this large dataset we procured an optimized set of partitions with high phylogenetic signal for resolving metazoan relationships. This optimized data set is amenable to the most appropriate and computationally intensive analyses using site-heterogeneous models of sequence evolution. We also employed several strategies to examine the potential for long-branch attraction to bias our inferences. Our analyses strongly support the Ctenophora as the sister lineage to other Metazoa. We find no support for the traditional view uniting the ctenophores and Cnidaria. Our findings are supported by Bayesian comparisons of topological hypotheses and we find no evidence that they are biased by long-branch attraction.

**Conclusions:**

Our study further clarifies relationships among early branching metazoan lineages. Our phylogeny supports the still-controversial position of ctenophores as sister group to all other metazoans. This study also provides a workflow and computational tools for minimizing systematic bias in genome-based phylogenetic analyses. Future studies of metazoan phylogeny will benefit from ongoing efforts to sequence the genomes of additional invertebrate taxa that will continue to inform our view of the relationships among the major lineages of animals.

**Electronic supplementary material:**

The online version of this article (doi:10.1186/s12864-015-2146-4) contains supplementary material, which is available to authorized users.

## Background

Advances in sequencing technology have led to a revolution in genomics, where draft genome assemblies for most species can be obtained at relatively little cost. One of the most significant outcomes anticipated of this revolution is an understanding of the interrelationships of the major lineages of multicellular animals, the Metazoa. A robust phylogeny for Metazoa will provide evolutionary context for understanding the timing and origins of the major features of animals including nervous systems [1], immune systems [2], cell types [3] and other complex traits. This phylogenetic framework will also impart important insights into the role that convergence could play in the evolution of such traits. Here we approach the question of metazoan relationships by extracting the phylogenetic signal present in a novel dataset derived from 36 publicly available whole genome sequences. The size of our dataset allows us to focus on identifying and ameliorating potential sources of bias that could stem from the inclusion of long-branch taxa or from data partitions with specific bias-inducing properties. We also explore signal between different modes of phylogenetic analysis and assess support for specific alternative hypotheses that are the current focus of debate in metazoan phylogenetics.

To date, numerous studies have applied large sequence datasets, drawn mostly from transcriptome sequencing efforts, to the problem of metazoan phylogeny [4–9]. Such approaches have yielded several important findings in recent years, most notably the position of the comb jellies (phylum Ctenophora) as sister to all remaining metazoan phyla including sponges (phylum Porifera). This surprising finding has attracted much attention because it suggests that neurons and other complex traits, present in ctenophores and eumetazoans but absent in sponges or placozoans, either evolved twice in Metazoa or were independently, secondarily lost in the lineages leading to sponges and placozoans [1, 10–12]. This relationship was first suggested by phylogenetic analyses of transcriptome datasets [4] and later by similar analyses that were augmented by whole genome sequences of two additional ctenophore species; *Mnemiopsis leidyi* [10] and *Pleurobrachia bachei* [1]. However, this finding is controversial and several other studies have argued that the basal position of ctenophores could be the result of long-branch attraction (LBA) or other artifacts stemming from noise present in large alignments [6, 7, 13].

While transcriptome-enabled phylogenetic analyses have doubtlessly proven powerful in the fabrication of large datasets representing large numbers of taxa, several caveats to this approach deserve mention. First, transcriptome based phylogenetic datasets only include data from genes that are expressed in the tissue collected for a given taxon. While whole organism transcriptome datasets are possible for small organisms, many taxa can only be represented by transcriptomes derived from selected tissues. Because different tissues may express different paralogs with distinct evolutionary histories, inaccuracies in the assessment of orthologous groups across taxa could result from this approach. The incomplete nature of transcriptome data is compounded when considering taxa with complicated life histories, which account for the majority of metazoan taxa. Second, transcriptome based data matrices are often sparse, consisting of much missing data, which can confound phylogenetic analyses [14, 15]. Finally, transcriptome datasets have been occasionally shown to include contaminants from other taxa, which could disrupt accurate phylogenetic reconstruction [13]. Whole genome sequences, while not without drawbacks of their own, do offer a solution to many of these issues encountered in transcriptome-based phylogenetic analyses.

The purpose of this study is to examine metazoan phylogeny with a focus on recent controversies surrounding the rooting of the animal tree and the position of the ctenophores using an alternative data set obtained exclusively from taxa with publicly available whole genome sequences. While previous studies of metazoan phylogeny included matrices derived from whole genome sequences [10, 16], the data set compiled here is by far the largest in terms of number of characters and taxa. Our novel data set is drawn from the gene models of 34 metazoan and two choanoflagellate genomes (Additional file 1: Table S1). We use a highly accurate orthology prediction procedure [17] followed by stringent alignment filtering to recover 1080 phylogenetically informative orthologous groups (OGs) that bear on metazoan phylogeny. We then assess a range of measures for each data partition including information content, saturation, rate of evolution, long-branch score, and taxon occupancy and explore how each of these characteristics impacts phylogeny estimation. We use these data to prepare a reduced set of partitions that fit an optimal set of criteria. This reduced matrix is amenable to the most accurate, but computationally intensive, analyses using site-heterogeneous models of sequence evolution [18]. Long branch attraction (LBA) has been suspected of influencing phylogenetic placement of several important metazoan lineages including the ctenophores [6, 7]. We employed several procedures to monitor the influence of LBA on our analyses. First we included several long-branch taxa with non-controversial phylogenetic positions, including the nematodes *Brugia* [19] and *Caenorhabditis* [20], the spider mite *Tetranychus* [21] and the larvacean tunicate *Oikopleura* [22] and monitored their positions in phylogenetic analyses. In addition, we tested the potential of outgroups and locus selection to induce topological artifacts. We find no evidence for LBA in any of our analyses. We also examined the possibility that specific categories of genes could support conflicting phylogenetic hypotheses but we find little evidence for a relationship between gene ontology and species topology. Finally, we examined the support for several competing alternative topologies pertaining to the position of the ctenophores by estimating and comparing their marginal likelihoods using stepping stone integration and Bayes factor analysis [23, 24].

In summary, we report analysis of the largest number of characters to be applied to metazoan phylogeny to date. We recover a phylogeny that is broadly consistent with the recent view of metazoan phylogeny [12]. All of the concatenated analyses and locus-selection experiments reported here support the hypothesis of the Ctenophora as sister to the other metazoan species. While support for this node varies depending on the subset of data analyzed, it is consistent across analyses and is strongly supported by the Bayesian test of topological hypotheses. Our results strongly reject the Coelenterata hypothesis that places cnidarians and ctenophores in a monophyletic group, or an arrangement placing sponges and ctenophores in a monophyletic group. Our study illustrates an optimized workflow for future analyses of hundreds or thousands of taxa represented by whole genome data and our user-friendly source code is freely available.

## Results and discussion

### Analyses of a large 1080-locus dataset supports *Ctenophora* as sister to remaining animal phyla

**Fig. 1:**
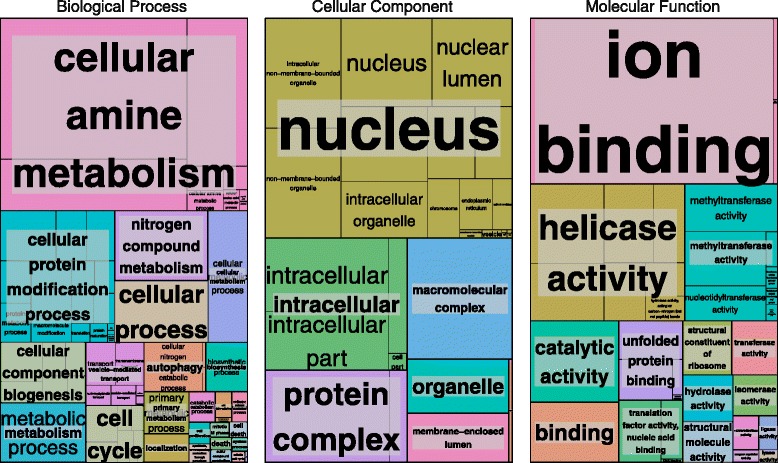
Visualization of gene ontology (GO) term enrichment across the total1080 dataset. In total, 142 GO terms were significantly enriched in the total1080 datasets compared to an outgroup reference annotation. Enrichments are depicted here for the Biological Process, Cellular Component and Molecular Function categories. In each, area subtended by a given GO term represents its frequency among significantly enriched GO terms

We retained 1080 individual alignments of putative orthologs following orthology prediction, removal of spurious sequences, alignment, and trimming (see Methods). In total, our data partitions are enriched for 142 gene ontology (GO) terms across the molecular function, cellular component, and biological process categories relative to a reference genome (Fig. 1). The alignments for these 1080 loci were concatenated into the ‘Total1080 matrix’ that consists of 385,669 amino acid positions at 75.85 % occupancy (Table 1).

**Table 1:** Characterization of matrices assembled for phylogenetic inference

Matrix name	Number of loci	Length (amino acids)	Missing data (%)	Criteria of locus selection
Total1080	1080	385,669	24.15	All loci that after trimming and filtering of paralogs
TaxaMin30	609	199,667	20.44	Loci with at least 30 taxa present
TaxaMin33	162	44,749	20	Loci with at least 33 taxa present
TaxaMin35	88	31,989	13.89	Loci with at least 35 taxa present
60Boot	55	37,682	25.92	Average locus tree bootstrap 60 or more
MareMatrix	143	35,030	20.02	MARE (Misof et al. 2013) algorithm filtering with alpha at 3.15
Slow108	108	33,580	18.91	10 % of the most slowly evolving loci
LowLB	171	60,397	22	Low LB scores in the outgroups, sponge, and ctenophore
Saturation108	108	36,954	19.78	10 % of the least saturated loci
Best108	108	41,808	15.55	10 % of loci scoring best in taxon occupancy, saturation, rate of evolution, average bootstrap, and LB scores
NoOutgr	143	35,022	18.49	As MareMatrix but realigned without *Monosiga* and *Salpingoeca* outgroups
NoAmphi	143	35,032	19.75	As MareMatrix but realigned without the sponge *Amphimedon*
NoOutgrAmphi	143	35,009	18.13	As MareMatrix but realigned without the sponge and the outgroups

We first inferred the topology from the Total1080 matrix under maximum likelihood (ML; [25, 26]) using best-fitting empirical models of protein evolution [27, 28] for each partition (Additional file 2: Figure S1). The topology of this tree reflects the emerging [1, 4, 5, 9, 10] but still controversial [6–8, 13] view of the ctenophores (*Mnemiopsis*) as the sister lineage to all other metazoans including sponges (*Amphimedon*). This topology also recovers all major metazoan clades and many widely-recognized relationships and the positions of several long-branch taxa, which include the nematodes *Brugia* and *Caenorhabditis*, the larvacean *Oikopleura* – by far the longest branch in our whole genome metazoan dataset – and the spider mite *Tetranychus*, are each as expected based on previously published studies [29–31].

### Analyses of refined datasets under site-heterogenous GTR-CAT model support *Ctenophora* as sister to remaining animal phyla

**Fig. 2:**
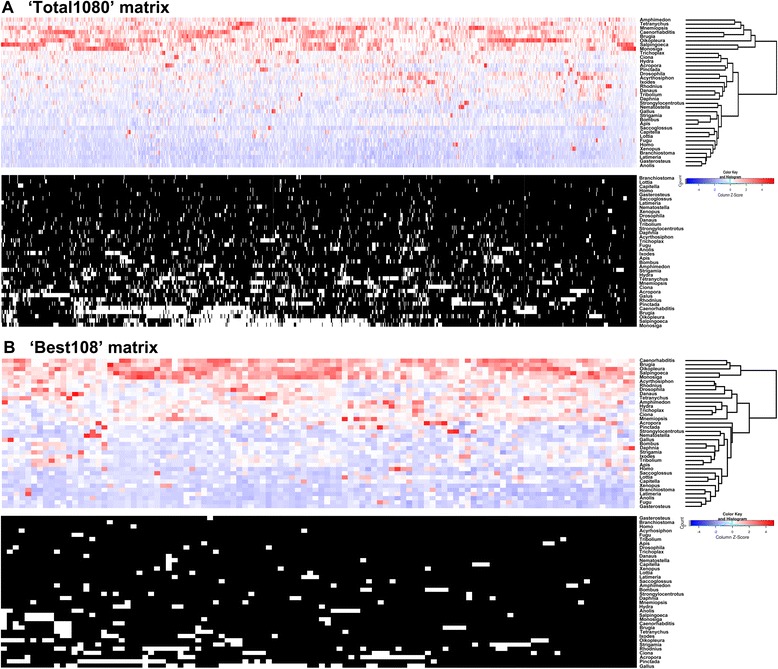
Distributions of long-branch scores and gene occupancy for Total1080 and Best108 matrices. In long-branch score heat maps, the scores were Z-scaled across columns to highlight among-taxon variability. Red indicates high long-branch scores relative to other taxa and blue denotes low scores. White in gene occupancy plots corresponds to missing data. The cladograms illustrate results of similarity by hierarchical clustering. Note that in the Total1080 dataset, *Amphimedon*, *Mnemiopsis and Tetranychus* cluster with other long-branched taxa that include the outgroups, the nematodes, and *Oikopleura*. However, in the Best108 matrix these taxa cluster with the main group, leaving only the outgroups, nematodes and *Oikopleura* in the long-branch cluster

The Total1080 dataset is too large to analyze under more appropriate, but computationally expensive site-heterogeneous models [18]. Because of this, we first analyzed each partition separately in order to derive data on 1) information content [32, 33], 2) taxon occupancy, 3) saturation [34], 4) long-branch score [35], and 5) rate of evolution. We then used these measures to select a set of 108 loci, 10 % of the total matrix that scored best across these criteria. This ’Best108’ matrix is amenable to computationally intensive analyses and consists of 41,808 amino acid positions at 84.45 % occupancy. We also assessed the influence of each of these criteria on the phylogeny, as they have each been proposed to negatively impact phylogenetic inference [13, 35, 36] (Additional file 3: Figure S2 and Additional file 4: Figure S3). Heat maps depicting long-branch scores among partitions for both the Total1080 and Best108 matrices and the taxon occupancy for each matrix are shown in Fig. 2. As with the Total1080 matrix, we performed partitioned ML inference under best-fitting empirical models of protein evolution on the Best108 matrix. In addition, we performed Bayesian analyses under site-heterogeneous CAT-GTR model [18].

The maximum likelihood (ML) trees for the Best108 matrix and the Total1080 ML tree show similar branching patterns. In both, the ctenophore *Mnemiopsis* is the sister to all other Metazoa with maximum bootstrap support and the centipede *Strigamia* is the sister to the chelicerates *Ixodes* and *Tetranychus*. The latter relationship reflects the Paradoxopoda hypothesis [37] but is only weakly supported by bootstrap values (67 %) in the Best108 tree. We note that recent studies [38] have demonstrated that this topology (Paradoxopoda) can result from model inadequacies in phylogenetic reconstruction under ML (see discussion).

**Fig. 3:**
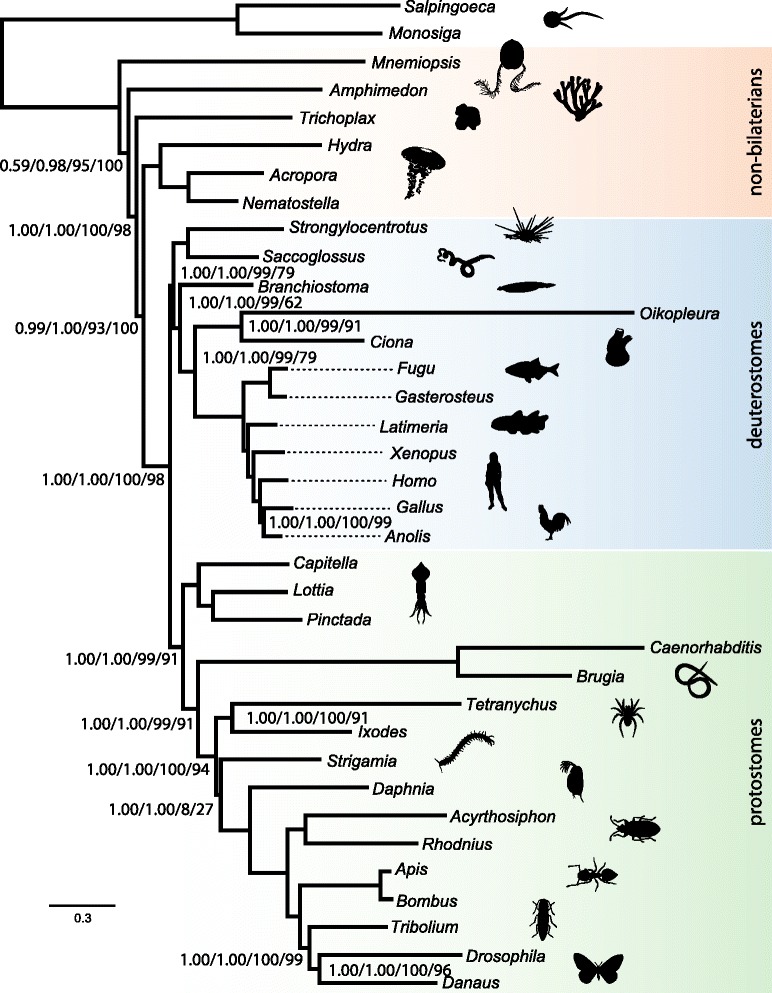
Summary of phylogenetic results. Tree topology and branch lengths are derived from the Best108 matrix data set analysis under CAT-GTR. Support values represent: posterior probabilities from PhyloBayes [66] analysis of Best108 matrix under CAT-GTR/posterior probabilities from PhyloBayes analysis of MareMatrix matrix under CAT-GTR/bootstrap support in Total1080 matrix under a partitioned empirical model/108-locus jackknife from the 1080 locus set. Unannotated nodes have maximum support for all measures. Scale bar in substitutions per site. Silhouettes from http://phylopic.org. For image attributions see Additional file 8

Bayesian analyses of the Best108 matrix under the CAT-GTR model produced a topology similar to the ML analysis of the same dataset, with the exception that the position of the centipede *Strigamia* is now resolved with maximum support as the sister to Pancrustacea, reflecting the Mandibulata hypothesis [38, 39]. This finding presumably reflects the more accurate fit of the model to the data, compared to ML analyses. Mandibulata is recovered with maximum support by analyses of all data subsets conducted under CAT-GTR (Fig. 3 and Additional file 4: Figure S3).

Similarly, all analyses conducted under CAT-GTR recovered the ctenophore as sister to the remaining Metazoa, but with varying degrees of support depending on the choice of dataset. The ‘60Boot’ dataset, comprised of partitions with average bootstrap support greater than 60, recovered ctenophores as sister other animal taxa with a posterior probability (PP) of 0.88; the matrix assembled using MARE [33] recovered this topology with PP = 0.98; and the Best108 dataset recovered this topology with PP = 0.59 (Fig. 3 and Additional file 4: Figure S3).

The CAT-GTR model accounts for differences in the substitution process across sites in a data set, but it does not account for compositional heterogeneity across branches. This among-branch heterogeneity is present in metazoan alignments from phylogenomic data [40] and may also negatively impact phylogeny estimation [41, 42]. Current implementations of models combining site- and branch-heterogeneity of substitution process are difficult to apply to large data sets [40, 43]. We therefore used an alternative approach that has been shown to be successful in reducing the effects of across-taxon heterogeneity [40] and recoded the amino acids in our Best108 matrix into six, four, and two categories and analyzed these recoded Best108 datasets under Bayesian CAT-GTR. Unfortunately, recoding data into fewer than the original 20 categories results in significant loss of signal in the alignments. The topologies resulting from these analyses where highly inconsistent placing *Trichoplax* as the sister to the remaining Metazoa, and in some cases, failing to recover a monophyletic Deuterostomia (Additional file 4: Figure S3).

### No evidence for long-branch attraction in Best108 dataset

The position of ctenophores as sister to the remaining Metazoa was recovered in most analyses above, but some workers have suggested that this topology can be explained by long-branch attraction (LBA), a phenomenon that causes long-branched taxa to group together artifactually in a phylogeny, often with strong support [44]. LBA is particularly common in datasets with poor taxon sampling or distant outgroups, in which fast evolving ingroup taxa can be ‘pulled’ to the base of the tree by long-branched outgroups. Several studies have indicated that LBA is a potential problem for reconstructing deep animal phylogeny [6–8, 13, 45]. In order to address the potential for LBA to bias our results, we explored various strategies to detect the LBA problem [44].

To test the possibility that the choanoflagellate outgroups affect non-bilaterian relationships through LBA, we assembled three matrices that excluded the choanoflagellate outgroups and/or the sponge *Amphimedon*. If the outgroups were to influence the branching order of non-bilaterians, we would expect the internal topology, or the support therein, to be impacted in an analysis excluding the outgroup taxa. Without the outgroups we lose the ability to reliably root the tree, but it is still possible to explore alternative rooting scenarios and to ask if the topology of the ingroup tree is different from those recovered in outgroup rooted analyses. For example, these analyses allow for the examination of possible rooting scenarios where ctenophores are sister to cnidarians, a hypothesis representing the so-called Coelenterata hypothesis [6, 8].

**Fig. 4:**
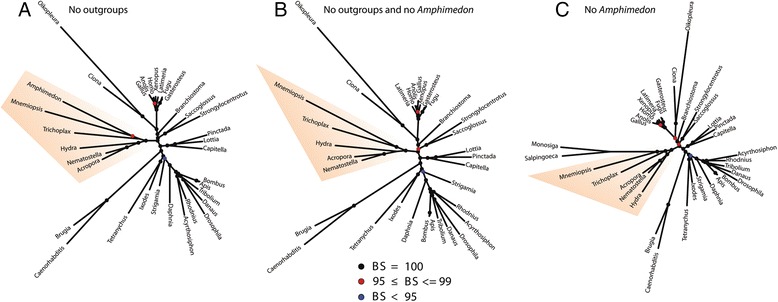
Summary of tests for Long Branch Attraction. Unrooted trees from analyses excluding putative long-branch taxa are shown. All analyses were conducted under maximum likelihood, partitioned empirical models. **a** Tree inferred without outgroups. **b** Tree inferred without outgroups and the sponge *Amphimedon*. **c** Tree inferred without the sponge *Amphimedon*. Black circles indicate bootstrap support of 100 % from 1000 replicates, red circles indicate support of 95 to 99 % and blue circles indicate support of 95 % or less. Non-bilaterian metazoan taxa are highlighted

We performed partitioned maximum likelihood analysis on the 1) ingroup/metazoan-only dataset, 2) a dataset where *Amphimedon* was removed and 3) a dataset where both *Amphimedon* and the outgroups were removed (Fig. 4). The ingroup-only topology derived from partitioned ML analysis allows for no possible rooting that would place ctenophores and cnidarians together in a monophyletic group (Fig. 4a). The sponge-ctenophore bipartition receives 98 % bootstrap support, which compares to 100 % for all other bipartitions in the tree, except the position of *Strigamia*, which is found in 94 % of bootstrap trees and the sister relationship of *Anolis* and *Gallus* at 99 % (Fig. 4a). If rooted with *Mnemiopsis*, the topology of this tree would be identical to the tree resulting from the ML analysis of a matrix that included outgroups (Additional file 2: Figure S1). If the position of ctenophores was affected by long-branch attraction, we would expect that the removal of outgroup taxa would alter the branching order or lessen support for non-bilaterian relationships [6]. Neither of these possibilities is evident in this analysis. We also examined topologies from partitioned ML analyses in which either the sponge *Amphimedon* (Fig. 4c), or both *Amphimedon* and the choanoflagellate outgroups were removed (Fig. 4b), and both show a similar pattern. A rooting where *Mnemiopsis* forms a clade with cnidarians, thus supporting the Coelenterata hypothesis, is not possible in any of these analyses.

### Different classes of genes tell similar stories

**Fig. 5:**
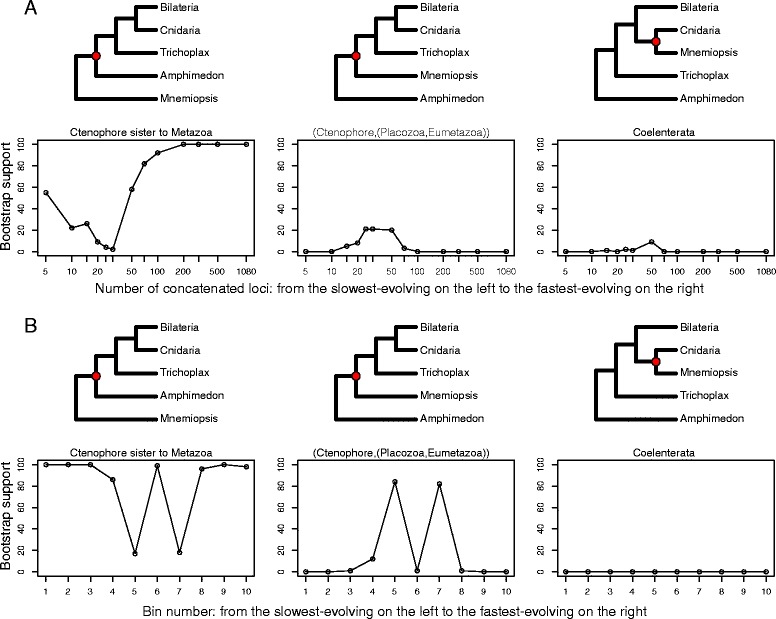
Sensitivity analyses using progressive concatenation and rate binning. **a** Support for alternative phylogenetic hypotheses under progressive concatenation from the slowest evolving to the fastest evolving loci. The x-axis represents number of loci concatenated in order of rate of evolution, from 5 of the most slowly evolving at left to all 1080 loci at right. The y-axis indicates bootstrap support. Red circles in cladograms above corresponding plots denote the node for which bootstrap support was assessed. **b** Support for alternative phylogenetic hypotheses across the data. The x-axis represents bin number and the y-axis indicates bootstrap support. Bin number 1 contains 108 slowest evolving loci in the data set and bin number 10 contains 108 fastest evolving loci. Red circles in cladograms above corresponding plots denote the node for which bootstrap support was assessed

Other studies focusing on metazoan phylogeny have suggested that the phylogenetic signal needed to resolve deep relationships is confined to slowly-evolving loci and that specific classes of genes may introduce noise that could mislead analyses [8, 31]. In order to explore the influence of rate of evolution of partitions on the support for metazoan relationships, we ranked all loci according to their rate of evolution, approximated by the average branch length of the ML tree inferred for each locus. We then performed a series of unpartitioned ML analyses on matrices that we generated of varying lengths, from few to all loci, beginning with the slowest evolving partitions then progressively adding faster and faster evolving partitions. Unpartitioned ML analysis was conducted for each iteration and support for topologies was assessed using 200 bootstrap replicates. Results from this progressive concatenation approach are detailed in Fig. 5a.

We evaluated support for several possible hypotheses on the position of the ctenophores in metazoan phylogeny including:

1. Ctenophora sister to Metazoa: ((*Mnemiopsis* (*Amphimedon,* other Metazoa))
2. Ctenophora sister to Eumetazoa: (*Mnemiopsis* (Placozoa, Eumetazoa))
3. The Coelenterata hypothesis, ctenophore as sister to Cnidaria:
  (*Mnemiopsis* (*Hydra*, *Nematostella*, *Acropora*))

Our progressive concatenation analyses show that support for the hypothesis of ctenophores as sister to Metazoa increases rapidly after the addition of greater than 30 partitions and bootstrap support for the hypothesis of ctenophore as sister to Metazoa increases to and is maintained at 100 % after 200 loci (Fig. 5a).

Next we explored phylogenetic signal in non-overlapping bins of concatenated data, also of increasing rates. We used a bin size of 108 loci, 10 % of the total dataset, per bin (Fig. 5b). In these analyses, support for the placement of ctenophores as sister to all remaining Metazoa is evident (86–100 % of bootstrap trees) in eight out of the ten bins, but was low in bins five and seven (17 and 18 %, respectively). We note that the three bins containing the most slowly evolving loci support the hypothesis that the ctenophore is the sister to other Metazoa. The most prevalent competing topology places the sponge *Amphimedon* as sister to all other metazoans, with ctenophores branching second (Ctenophore (Placozoa, Eumetazoa)). None of the analyses showed consistent support for the Coelenterata hypothesis.

Our selection of a bin size of 108 loci per bin permitted statistical analyses of GO term enrichment on a bin-by-bin basis. However, these analyses did not reveal a single instance of GO term enrichment in any of the bins compared to the GO terms present in the total dataset. While individual bins may differ in their rates of evolution and the topologies they support, their composition is not significantly different from the total 1080 matrix as measured by GO term enrichment analyses. To further explore the effect of GO category on phylogenetic signal, we prepared datasets for phylogenetic analysis from the only two GO categories from our initial 1080 gene dataset (Fig. 1) that contained greater than 30 loci: the mitochondrial cellular component and the nuclear cellular component. Concatenated analyses of these datasets under ML produced similar results, however the tree estimated for the mitochondrial cellular component dataset was generally poorly supported (Additional file 5: Figure S4).

### Bayesian tests of topological hypotheses strongly support Ctenophora as sister to remaining Metazoa

Both ML partitioned analyses under the best fitting models and Bayesian analyses under GTR-CAT supported the hypothesis of ctenophores as sisters to the remaining metazoans. Next we sought to understand the relative degree of support for this hypothesis compared to other alternatives. Bayesian tests of topological hypothesis are a powerful means of estimating the relative support for conflicting topologies [23]. We estimated the marginal likelihoods of three possible hypotheses of monophyly that relate to the position of the ctenophores in our dataset using stepping stone integration [24] including:

1. Monophyly of Porifera and Eumetazoa to the exclusion of Ctenophora
2. Monophyly of Ctenophora and Eumetazoa to the exclusion of Porifera
3. Monophyly of Ctenophora and Cnidaria to the exclusion of all other taxa

Our results indicated very strong support for proposal #1 above, which represents the hypothesis of ctenophores as the sister to remaining Metazoa. In addition, all other hypotheses of monophyly were strongly rejected (Table 2).

**Table 2:** Bayes factor comparisons of hypotheses relating to the position of the Ctenophores

Hypothesis	Marginal Likelihood For	Marginal Likelihood Against	Log Units Diff/2ln B_10_	Interpretation^a^
Monophyly of Porifera and Eumetazoa to the exclusion of Ctenophora	−1513039.43	−1513272.74	233.31/10.9	Very Strongly Supported
Monophyly of Ctenophora and Eumetazoa to the exclusion of Porifera	−1513039.63	−1513180.01	79.01/8.7	Strongly Rejected
Monophyly of Ctenophora and Cnidaria to the exclusion of all other taxa	−1514055.49	−1513062.25	993.24/13.6	Very Strongly Rejected

^a^Interpretation from [23]

## Conclusions

### A large dataset for the estimation of metazoan phylogeny provides an alternative line of support for the new view of animal phylogeny

Large data sets are often insufficient to resolve recalcitrant nodes in the animal tree of life and it has long been recognized that simply increasing the amount of data can exacerbate systematic bias in phylogeny estimation [13, 45, 46]. Because of this, two approaches to improving phylogenomic inference have been proposed. One focuses on the quality of the data and attempts to select only the ‘best’ characters or loci for analysis based on various characteristics [32, 33, 35]. The other is to employ more realistic models of sequence evolution that account for various systematic biases [13, 15, 45]. Here we leverage both approaches and, due to the large size of our initial data matrix, we are able to minimize the impact of various sources of non-phylogenetic signal while retaining a large number of characters for analysis.

Our Best108 dataset represents a refinement of the Total1080 dataset as shown in Fig. 2 where differences in long-branch score and taxon occupancy between the two datasets are compared. In both datasets, hierarchical clustering sorts a subset of taxa into a long-branch group of sequences. In the Total1080 matrix, this long-branch cluster includes eight taxa including *Mnemiopsis* and *Amphimedon*. In the Best108 matrix, the long-branch cluster is reduced to five taxa and only includes those taxa that reside in non-controversial positions (e.g. both choanoflagellate outgroups, both nematodes and the larvacean *Oikopleura*) and these positions are recovered in all analyses of the Best108 dataset. In addition, taxon occupancy is enhanced in the Best108 dataset over the Total1080 dataset, while the rates of evolution are lower and the potential for saturation is minimized. For these reasons, we expect that the reduced dataset should contain less phylogenetic noise than the Total1080 dataset.

### Strong support for the Ctenophora as the evolutionary sister to other metazoans

Our results are congruent with several recent studies [1, 4, 9, 10] that depict the ctenophores as the sister lineage to all other metazoans. This hypothesis receives maximum support in all of our ML analyses (Fig. 3; Additional file 3: Figure S2) and is supported in most Bayesian analyses conducted under the more parameter-rich CAT-GTR model (Fig. 3; Additional file 4: Figure S3) with the exception of recoded datasets that attempted to control for among-taxon rate heterogeneity but failed to recover several well-accepted clades (Additional file 4: Figure S3). Further, our additional analyses suggest that long-branch attraction artifacts do not drive this result (Fig. 4; see also Whelan et al. [9]) and it is supported by progressive concatenation analyses (Fig. 5). Perhaps most compelling are our tests of competing hypotheses for the position of the ctenophores using Bayes factors. This approach to topology comparison is more robust to statistical error than common ML procedures, and the analyses presented here were done using stepping stone integration, which is the most accurate method of estimating the marginal likelihoods of competing hypotheses currently available [24]. Our comparisons of the marginal likelihoods of each plausible hypothesis for monophyly that could explain the position of the ctenophores in animal phylogeny lend very strong support for the hypothesis of ctenophores as the sister lineage to remaining Metazoa, while strongly or very strongly [23] rejecting other competing hypotheses (Table 2).

Our results are consistent with the Parahoxozoa hypothesis, which postulates a single origin of Hox genes in the clade comprised of Bilateria, Cnidaria and Placozoa, to the exclusion of Porifera and Ctenophora [1, 10, 47]. None of our analyses support the Coelenterata hypothesis uniting Cnidaria and Ctenophora, a clade that has been recovered in some morphological and phylogenomic analyses [6, 8, 48, 49].

### Consistency of results under different models for molecular evolution

Our results relating to the position of ctenophores are consistent across the majority of analyses, but one taxon, the sole representative myriapod *Strigamia*, is decidedly the most labile across analyses. While Paradoxopoda (chelicerates plus myriapods) receives support in bootstrap replicates of the concatenated Total1080 data set (92 %, Additional file 3: Figure S2), support for this clade varies drastically across analyses (Fig. 3). Paradoxopoda is supported in most ML trees, but Mandibulata (pancrustaceans plus myriapods) is strongly preferred in a subset of these analyses and in most of the Bayesian analyses conducted under CAT-GTR (Additional file 3: Figure S2 and Additional file 4: Figure S3). The instability of *Strigamia* is further demonstrated in progressive concatenation analyses (Additional file 6: Figure S5). Our findings are consistent with previous studies that demonstrate the importance of model selection and the potential for LBA artifacts in the placement of the myriapod lineage [38]. In contrast to ctenophores where their position is invariable across the models of molecular evolution employed, the position of the myriapods appears to be sensitive to model selection.

### Concluding remarks

Our study addresses the problem of basal metazoan relationships using a large dataset drawn exclusively from whole genome sequences. By applying stringent filtering procedures on a very large initial dataset, we were able to obtain reduced datasets that are still much larger than previous analyses, but are exclusively comprised of partitions with high taxon occupancy and low potential for non-phylogenetic signal. Ctenophores are strongly supported as the sister to the remaining Metazoa and support for Parahoxozoa is overwhelming in our analyses, arguing against the traditional grouping of ctenophores and cnidarians into Coelenterata. The term “Coelenterata” has been associated with numerous meanings throughout the history of invertebrate zoology and dates to at least the 19th century. While consistently referring to a group that includes Cnidaria and Ctenophora, various workers have also included echinoderms, bryozoans, tunicates and sponges in different formulations of Coelenterata (reviewed in Hyman [50]). Our results are consistent with several recent studies that strongly reject the systematic utility of the term, finding coelenterates (animals with a central, fluid-filled cavity) to be a polyphyletic assemblage.

One obvious drawback of exclusively relying on taxa with whole genome sequences for metazoan phylogeny reconstruction is that taxon sampling is necessarily low compared to other studies that have analyzed transcriptome-based datasets. While numerous workers have emphasized the importance of taxon sampling [4, 13], others have emphasized the importance of data matrix size [51]. Ideally, both parameters would be maximized while maintaining the computational tractability of matrices under the most appropriate models for molecular evolution. Indeed, even the Best108 dataset and its limited taxonomic sample makes conducting all of the analyses presented here under Bayesian CAT-GTR computationally intractable (We estimate that 125 years of single-core computation time in total was expended in the present study).

Future studies of metazoan phylogeny will benefit from ongoing efforts to sequence the genomes of additional invertebrate taxa that will inform our view of the relationships between the major lineages of animals [52]. This is true especially of sponges, where branches subtending this group could be dramatically shortened [1, 6, 9] with additional sampling. More genomic resources coupled with better methods that account for systematic biases [15] and the use of additional characters such as presence/absence of genes [10] could soon provide us with a robust phylogeny including all major metazoan lineages [4, 53].

## Methods

### Taxon sampling and data acquisition

Taxon sampling aimed to maximize the phylogenetic breadth of species that can inform metazoan relationships, while relying exclusively on species with whole genome sequences. Long-branch attraction (LBA) has been suspected in contributing to the placement of the ctenophores in metazoan phylogeny [7]. We specifically included other known long-branched taxa such as the nematodes *Brugia* and *Caenorhabditis*, the tunicate *Oikopleura,* and the spider mite *Tetranychus* so that we could monitor the potential for LBA in our dataset. Additional file 1: Table S1 lists these species and the genome databases from which they were obtained.

### Orthology prediction, alignment trimming, and removal of spurious sequences

Gene orthology analysis was performed using a pre-release version 2.0 of the OrthologID pipeline [17]. This version of OrthologID uses the MCL algorithm [54, 55] for improved clustering and includes automated extraction of orthologs from gene trees into a partitioned matrix. Amino acid sequences of 1,047,986 gene models from the complete gene sets of all 36 species were used as input to OrthologID, which produced 26,612 orthologous groups with at least 4 species represented. We then selected partitions that included 27 taxa or more for inclusion in our analyses, resulting in a total of 1162 orthologous groups (OGs). OGs were aligned in MUSCLE [56] using the default settings and trimmed with trimAl v1.4 [57] using the *-resoverlap 0.5* and *-seqoverlap 50* settings that remove taxa with relatively poor sequence representation within each alignment, followed by *-gappyout* algorithm trimming that removes gap-rich columns. We then conducted maximum likelihood (ML) tree estimation on each locus (see below). We identified potentially spurious sequences with terminal branches more than five times longer than the average for the tree. We discarded 211 individual sequences using this arbitrary cut-off. We also discarded partitions that had more than 40 % missing data. This resulted in a set of 1080 curated loci used for further analyses and construction of the ‘Total1080’ data matrix.

### Gene ontology analyses

One randomly chosen gene from each of 1080 OGs was subjected to blast, annotation and mapping using Blast2GO [58]. Gene Ontology identification numbers (GO IDs) for each Metazoan partition were abstracted from this analysis and tested for enrichment against GO IDs from the genome of *Arabidopsis thaliana*, a taxon outside the phylogenetic scope of the focal taxa. Enrichment analyses were performed using Singular Enrichment Analyses and the Fisher’s Exact Test implemented in agriGO [59]. Enrichment analyses of GO terms between individual bins of metazoan orthologous groups and the total metazoan dataset were also performed using Singular Enrichment Analyses and the Fisher’s Exact Test implemented in agriGO [59] using GO IDs from the total set of 1080 OGs as a background annotation. Results were visualized using REVIGO [60]

### Single gene trees, locus selection and construction of the Best108 dataset

We used Phyutility [61] for concatenation of all multiple-gene matrices and MESQUITE v2.75 [62] to convert among file formats. In order to examine the individual topologies of partitions, we estimated a tree for each of the 1080 alignments using the best-fitting empirical model under maximum likelihood (ML) in RAxML [26]. We also performed 200 bootstrap replicates for each gene tree. The alignment and corresponding single-gene tree characteristics (see below) served as a basis for several alternative locus selection strategies. ML analyses of each for each concatenated dataset are reported in Additional file 3: Figure S2.

#### Locus selection based on information content

We assembled two matrices selecting for information content. One was a concatenation of loci with average nodal bootstrap support higher than 60 % (‘60Boot’) [32]. The other matrix was a result of MARE filtering [33] of the Total1080 data set (‘MareMatrix’). We conserved all 36 taxa and used alpha setting of 3.15 (3.00 is default, higher means smaller matrix and higher information content) to obtain a data set of size similar to that of ‘60Boot’ matrix.

#### Taxon occupancy and missing data

We concatenated the following matrices with varying levels of taxon representation: the Total1080 matrix that included all 1080 filtered and trimmed orthologous groups with at least 27 taxa represented, ‘TaxaMin30’ with 609 loci having at least 30 taxa, ‘TaxaMin33’ with 162 loci having at least 33 taxa, and ‘TaxaMin35’, a matrix comprising 88 loci with minimum taxon occupancy of 35 out of 36 total species.

#### Saturation

We evaluated saturation in each locus by performing simple linear regression on uncorrected p-distances against inferred distances for each locus [34]. In the absence of sequence saturation, the expectation is that these distances would show a perfect fit to simple linear regression. When there is a need of correction for multiple substitutions, however, the curve will depart from linearity. We used slope and R^2^ of the regression to assess fit in each locus.

#### Long-branch score

The so-called ‘long-branch score’ ([35]; LB score) makes it is possible to assess patterns of branch length distribution across the data. The score is a taxon-specific measure defined as the mean pairwise distance of a terminal to all other terminals, relative to average pairwise distance across all taxa. Because of its taxon-specificity, direct comparisons are not possible among loci, and Struck [35] suggested standard deviation of LB scores as a measure by which loci can be compared. However, we observed that alignments with low standard deviation of LB scores had high proportion of missing data for long-branched taxa. Because of this we implemented an alternative approach, focusing on LB scores of the long-branched *Amphimedon*, *Mnemiopsis*, and the outgroups, *Monosiga* and *Salpingoeca*. We first identified LB mode of density distribution for each taxon, calculated from the Total1080 data set. We then used the number (zero to four) of these focal taxa falling under the mode in each locus to rank all loci. We also concatenated a matrix with 171 loci (‘LowLB’ matrix) with low LB scores and at least three of the four species, therefore minimizing missing data for these target taxa.

#### Rate of molecular evolution

We used the average branch length of a tree as an approximation of the rate of evolution. The trees were derived from an ML analysis of each of the 1080 loci under the best-fitting empirical model of sequence evolution (see above). The average branch length was calculated by dividing the total tree length by the total number of edges (internal and terminal branches) in the tree. While this measure does not account for the differences in taxon sampling among the alignments, we found that it provides a useful estimate of relative rates among loci in this data set. A list of loci ranked by average branch length served as a basis for progressive concatenation and binned analyses. We also concatenated a matrix with 10 % of the most slowly evolving genes for a partitioned maximum likelihood analysis (‘Slow108’ matrix).

#### Construction of the Best108 matrix

We scored the loci by rank in each of the above characteristics (information content, taxon occupancy, saturation, rate of evolution, and long-branch score) and chose 108 (10 % of all loci) loci with the best scores to assemble the Best108 matrix. We used R packages *seqinr* [63] and *ape* [64] to compute these statistics, and our R script can be found in the Dryad repository and on GitHub (see Availability of supporting data below). It is well-annotated and allows custom input from the user’s alignments and gene trees. Results are also summarized for each locus in a supplementary table ‘gene_stats.xlsx’ available on Dryad.

### Maximum likelihood analyses of partitioned data sets

We used PartitionFinderProtein [65] to find optimal partitioning schemes and models for all concatenated matrices. Because PartitionFinder by default uses Neighbor Joining to estimate guide trees, we first inferred maximum likelihood trees for each unpartitioned matrix using RAxML and used these as user-supplied guide trees for PartitionFinder. We then used RAxML standard versions 8.1 and newer to infer a maximum likelihood tree with support drawn from 1000 rapid bootstrap replicates.

### Jackknife support in the ‘Total1080’ data set

Jackknife analyses were carried out for 300 replicates of 108-locus (10 %) and 900 replicates of 20-locus matrices randomly selected from the 1080 locus set with a custom Python script (http://github.com/marekborowiec/metazoan_phylogenomics/blob/master/phylo_jackknife.py). This program prompts for user input and allows for easy creation of locus-jackknife alignments with other data sets. Maximum likelihood trees were estimated for each unpartitioned matrix under the best empirical model selection scheme in RAxML.

### Bayesian analyses of concatenated datasets

For Bayesian inference, we used PhyloBayes MPI v1.5a [66], with CAT-GTR as the amino acid replacement model. Analysis with recoded amino acids were performed using PhyloBayes 3.3f [67]. We used three different recoding schemes, which recoded amino acids with six, four, and two groups corresponding to the “dayhoff6”, “dayhoff4”, and “hp” schemes for the *-recode* option in PhyloBayes. Two independent Monte Carlo Markov chains were produced for every matrix. The resulting tree for each matrix is the majority-rule consensus of all trees pooled across both chains sampled at equilibrium. Trace plots were generated using the *mcmcplots* package [68] in R. A summary of statistics from PhyloBayes analyses is given in Additional file 7: Table S2.

### Progressive concatenation and binned analyses

To assess the effect that partitions with high rates of evolution have on the inference, we also incrementally concatenated loci evolving at increasing rates. We sorted the 1080 gene partitions by their rates of evolution, and created ten matrices by concatenating 5, 10, 15, 20, 30, 50, 100, 200, 300, and 500 slowest evolving loci. We ran a 200-bootstrap replicate, unpartitioned RAxML search on all these matrices and the all-inclusive matrix of 1080 loci. We also performed binned analyses where loci were concatenated into ten 108-gene non-overlapping matrices and subjected to a RAxML search as the above. We then mapped bootstrap support for nodes in alternative topologies using RAxML for all progressively concatenated matrices and bins. The trees and support from these experiments can be found in the Dryad repository associated with this article.

### Estimating of marginal likelihoods using stepping stone integration

Tests of topological hypotheses were conducted in MrBayes 3.2 [69] using stepping stone integration [24] under default parameters. Briefly, we sampled from 50 steps with 5000 generations each. One step was discarded as burnin. Marginal likelihoods were estimated from 245,000 generations and interpreted as per [23]. Control files and output from stepping stone runs are included in the Dryad repository associated with this article.

## Availability of supporting data

The data sets supporting the results of this article are available in the Dryad repository, http://dx.doi:10.5061/dryad.k6tq2. Additionally, all computational scripts used in the work reported here are found at the GitHub repository, http://github.com/marekborowiec/metazoan_phylogenomics.git

These scripts, written in R and Python languages, have been well-annotated and allow for customized input. All sequence datasets, alignments, spreadsheets, annotation files, output files and lists of gene ontology terms for analysis are available at the Dryad link associated with this study. This supplementary data also includes details of all PartitionFinder, RAxML, PhyloBayes and MrBayes 3.2 analyses conducted.

## Additional files

Additional file 1: Table S1. List of genomes and URLs utilized in the study (PDF 38 kb) Additional file 2: Figure S1. Maximum Likelihood tree of the Total1080 dataset. (PDF 6608 kb) Additional file 3: Figure S2. Maximum Likelihood analyses of each type of filtered dataset. (PDF 2041 kb) Additional file 4: Figure S3. Bayesian CAT-GTR trees of each type of filtered and recoded dataset. (PDF 7912 kb) Additional file 5: Figure S4. Comparison between trees derived from loci with mitochondrial (A) or nuclear (B) expression (cellular components). (PDF 10799 kb) Additional file 6: Figure S5. Progressive concatenation and binning analysis of the position of *Strigamia*. (PDF 2880 kb) Additional file 7: Table S2. Summary of statistics from Bayesian searches of phylogeny using PhyloBayes. Maxdiff < 0.1: good run, maxdiff < 0.3: acceptable. (PDF 11 kb) Additional file 8:
**Image attributions.** (PDF 12 kb)

## Abbreviations

GO: Gene ontology
GTR: General time-reversible
LB score: Long-branch score
LBA: Long-branch attraction
ML: Maximum likelihood
OG: Orthologous group
PP: Posterior probability
