# Supplementary material for: Extracting phylogenetic signal and accounting for bias in whole-genome data sets supports the Ctenophora as sister to remaining Metazoa
Source: BMC Genomics. 2015 Nov 23;16:987. doi: 10.1186/s12864-015-2146-4 (PMC4657218; doi:10.1186/s12864-015-2146-4)
Supplement: Additional file 1: Table S1. — List of genomes and URLs utilized in the study (PDF 38 kb) [file 12864_2015_2146_MOESM1_ESM.pdf]

Organism	Classification	Reference	Protein Database
Acropora digitifera (stony coral)	Cnidaria: Anthozoa	Shinzato *et al.* 2011. Using the *Acropora digitifera* genome to understand coral responses to environmental change. Nature 476:320-323.	Okinawa Institute of Science and Technology, Predicted Proteins ver 1.0
Acyrthosiphon pisum (pea aphid)	Arthropoda: Hexapoda	International Aphid Genomics Consortium. 2010. Genome sequence of the pea aphid *Acyrthosiphon pisum*. PLoS Biol. 8:e1000313.	NCBI, PRJNA29489
Amphimedon queenslandica (desmosponge)	Porifera: Desmospongiae	Srivastava *et al.* 2010. The *Amphimedon queenslandica* genome and the evolution of animal complexity. Nature 466:720-726	JGI, Protein Assembly v 1.0
Anolis carolinensis (green anole)	Chordata: Vertebrata	Afoldi, J. et al. 2011. The genome of the green anole lizard and a comparative analysis with birds and mammals. Nature 477:587-91.	NCBI, PRJNA60547
Apis mellifera (honey bee)	Arthropoda: Hexapoda	Weinstock *et al.* 2006. Insights into social insects from the genome of the honeybee *Apis mellifera*. Nature 443:931-949.	NCBI, PRJNA13343
Bombus terrestris (buff-tailed bumble bee)	Arthropoda: Hexapoda	Sadd *et al.* 2015. The genomes of two key bumblebee species with primitive eusocial organization. Genome Biol. 16:76.	Bimp 2.o
Branchiostoma floridae (lancelet)	Chordata: Cephalochordata	Putnam *et al*. 2008. The amphioxus genome and the evolution of the chordate karyotype. Nature 453:1064-1071.	JGI, Protein Assembly v 1.0
Brugia malayi (filarial worm)	Nematoda	Ghedin *et al.* 2007. Draft Genome of the filarial nematode parasite *Brugia malayi*. Science 317:1756-1760.	UniProt
Caenorhabditis elegans (nematode)	Nematoda	Reboul *et al.* 2003. *C. elegans* ORFeome version 1.1: experimental verification of the genome annotation and resource for proteome- scale protein expression. Nature 34:35-41.	UniProt
Capitella telata (polycheate worm)	Annelida: Polychaeta	Simakov *et al.* 2012. Insights into bilaterian evolution from three spiralian genomes. Nature 493:526-531.	JGI, Filtered Models Protein Assembly v1.0
Ciona savignyi (sea squirt)	Chordata: Tunicata	Satoh *et al.* 2003. *Ciona intestinalis*: an emerging model for whole-genome analyses. Trends Genet. 19: 376-381.	Ensembl database, CSAV2.0.58
Danaus plexippus (monarch butterfly)	Arthropoda: Hexapoda	Zhan *et al.* 2011. The monarch butterfly genome yields insights into long-distance migration. Cell 147:1171-1185.	
Daphnia magna (water flea)	Arthropoda: Crustacea	Colbourne, J. *et al.* 2011. The ecoresponsive genome of *Daphnia pulex*. Science 331:555-561.	JGI, Filtered Models Protein Assembly v.1.1
Drosophila melanogaster (fruit fly)	Arthropoda: Hexapoda	Adams *et al*. 2000. The genome sequence of *Drosophila melanogaster*. Science 287:2185-2195.	Ensembl database, BDGP4.3.41
Gallus gallus (chicken)	Chordata: Vertebrata	Hillier *et al.* 2004. Sequence and comparative analysis of the chicken genome provide unique perspectives on vertebrate evolution. Nature 432:695-716.	Ensembl database, WASHUC1
Gasterosteus aculeatus (stickleback)	Chordata: Vertebrata	Jones *et al.* 2012. The genomic basis of adaptive evolution in threespine sticklebacks. Nature 484:55-61.	http://www.ensembl.org/Gasterosteus_aculeatus/Info/Index
Homo sapiens (human)	Chordata: Vertebrata	International Human Genome Sequencing Consortium. 2004. Finishing the euchromatic sequence of the human genome. Nature 431:931-45.	NCBI, PRJNA168
Hydra magnipapillata (hydrozoan)	Cnidaria: Medusozoa	Chapman *et al*. 2010. The dynamic genome of *Hydra*. Nature 464:592-596.	NCBI, PRJNA31231
Ixodes scapularis (black-legged tick)	Arthropoda: Arachnida	Megy, K. *et al.* 2012. VectorBase: improvements to a bioinformatics resource for invertebrate vector genomics. Nucleic Acids Res. 40:D729-D734.	Vector Base, IscaW1.2
Latimeria chalumnae (coelacanth)	Chordata: Vertebrata	Amemiya *et al.* 2013. The African coelacanth genome provides insights into tetrapod evolution. Nature 496:311-316.	http://www.ensembl.org/Latimeria_chalumnae/Info/Index
Lottia gigantea (owl limpet)	Mollusca: Gastropoda	Simakov *et al.* 2012. Insights into bilaterian evolution from three spiralian genomes. Nature 493:526-531.	JGI, Filtered Models Protein Assembly v1.0
Mnemiopsis leiydei (comb jelly)	Ctenophora	N/A	NHGRI Mnemiopsis Genome Project
Monosiga brevicollis (choanoflagellate)	Protozoa: Choanoflagellata	King *et al.* 2008. The genome of the choanoflagellate. Nature 45:783-788.	JGI, "Best Proteins" Monbr1
Nematostella vectensis (sea anemone)	Cnidaria: Anthozoa	Putnam *et al*. 2007. Sea anemone genome reveals ancestral eumetazoan gene repertoire and genomic organization. Science 317:86-94.	JGI, Protein Assembly v 1.0
Okopleura dioica (larvacean)	Chordata: Tunicata	Seo *et al.* 2001. Miniature genome in the marine chordate *Oikopleura dioica*. Sci	http://www.genoscope.cns.fr/externe/Download/Projets/Projet_HG/data/
Pinctada fucata (pearl oyster)	Mollusca: Bivalvia	Takeuchi *et al*. 2012. Draft genome of the pearl oyster *Pinctada fucata*: A platform for understanding bivalve biology. DNA Res. 19:117-130.	Okinawa Institute of Science and Technology, Predicted Proteins ver 1.0
Rhodnius prolixus (kissing bug)	Arthropoda: Hexapoda	Megy, K. *et al.* 2012. VectorBase: improvements to a bioinformatics resource for invertebrate vector genomics. Nucleic Acids Res. 40:D729-D734.	VectorBase, RproC1.0
Saccoglossus kowalevskii (acorn worm)	Hemichordata	Gerhart *et al.* Sequencing the genome of the hemichordate *Saccoglossus kowalevskii*.	http://www.hgsc.bcm.tmc.edu/content/acorn-worm-genome-project
Salpingoeca rosetta (choanoflagellate)	Protozoa: Choanoflagellata	Rairclough *et al.* 2012. Premetazoan genome evolution and the regulation of cell differentiation in the choanoflagellate *Salpingoeca rosetta.* Genome Biol. 14:R15	https://www.broadinstitute.org/annotation/genome/multicellularity_project/GenomeDescriptions.html
Strigamia maritima (centipede)	Arthropoda: Myriapoda	Patel *et al*. Ecdysozoan sequencing proposal.	http://www.strigamia-annotation.org/cap
Strongylocentrotus purpuratus (sea urchin)	Echinodermata	Sea Urchin Genome Sequencing Consortium, et al. 2006. The genome of the sea urchin Strongylocentrotus purpuratus. Science 314:941-952.	BCM
Takifugu rubripes (pufferfish)	Chordata: Vertebrata	Aparicio *et al.* 2002. Whole-genome shotgun assembly and analysis of the genome of *Fugu rubripes*. Science 297:1301-1310.	Ensembl database (whatever version preceded the current one on the site)
Tetranychus urticae (spider mite)	Arthropoda: Arachnida	Grbic et al. 2011. The genome of Tetranychus urticae reveals herbivorous pest adaptations. Nature 479:487-492.	http://metazoa.ensembl.org/Tetranychus_urticae/Info/Index
Tribolium castaneum (flour beetle)	Arthropoda: Hexapoda	Richards *et al*. 2008. The genome of the model beetle and pest *Tribolium castaneum*. Nature 452:949-955.	NCBI, PRJNA12540
Trichoplax adhaerens (placazoan)	Placozoa	Srivastava M. *et al*. 2008. The *Trichoplax* genome and the nature of placozoans. Nature 454:955-60.	JGI, Protein Assembly v 1.0
Xenopus tropicalis (Western clawed frog)	Chordata: Vertebrata	Hellsten *et al*. 2010. The genome of the Western clawed frog *Xenopus tropicalis*. Science 328:633-636.	JGI, Filtered Models Version 4.0 or 4.1 ?
