# Supplementary figures and images for: Extracting phylogenetic signal and accounting for bias in whole-genome data sets supports the Ctenophora as sister to remaining Metazoa
Source: BMC Genomics. 2015 Nov 23;16:987. doi: 10.1186/s12864-015-2146-4 (PMC4657218; doi:10.1186/s12864-015-2146-4)

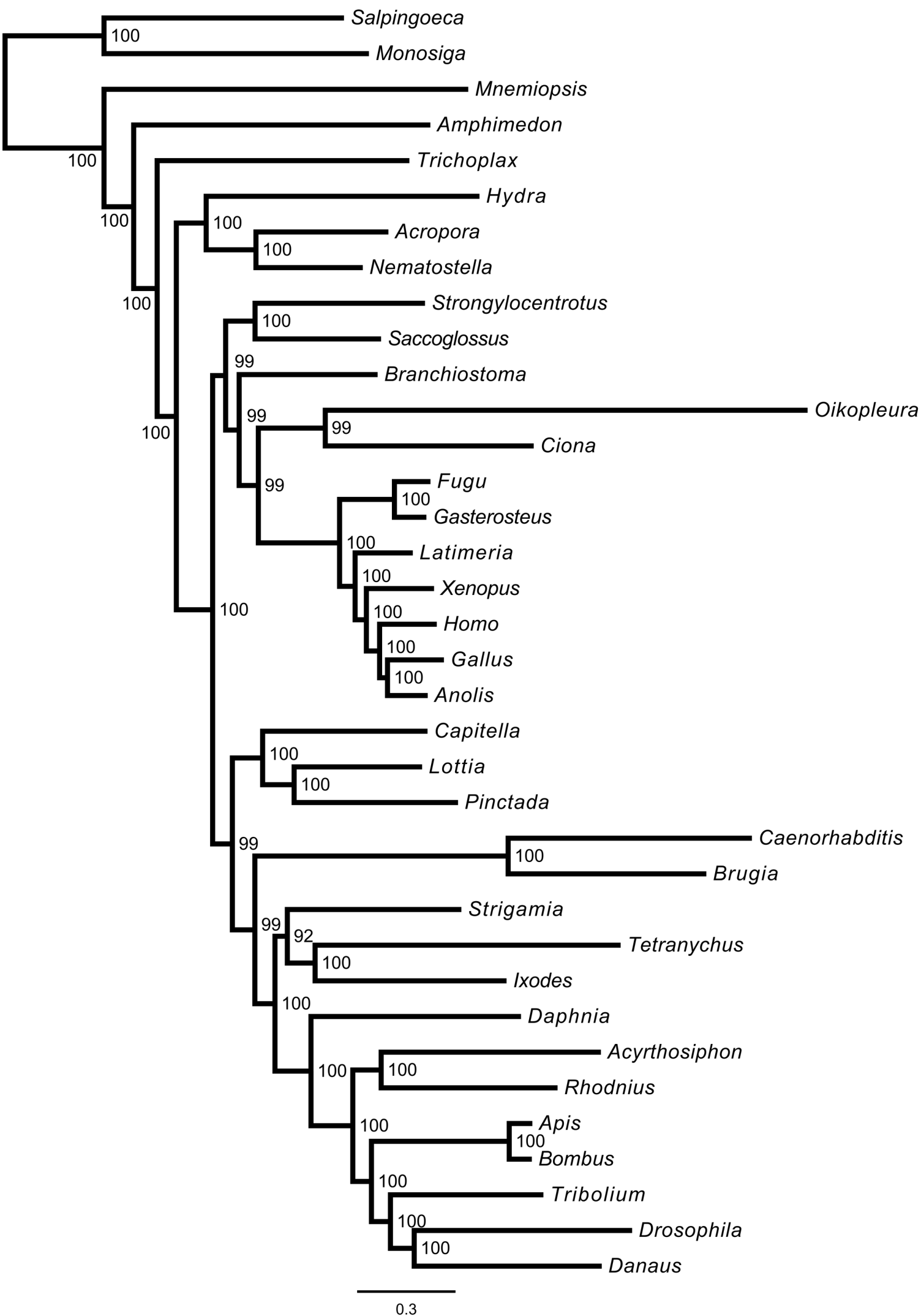

Supplement: Additional file 2: Figure S1. — Maximum Likelihood tree of the Total1080 dataset. (PDF 6608 kb) [file 12864_2015_2146_MOESM2_ESM.pdf]

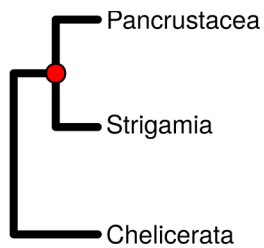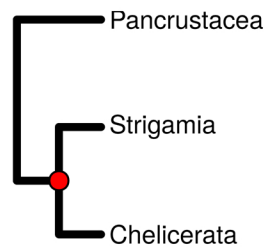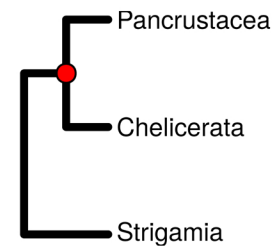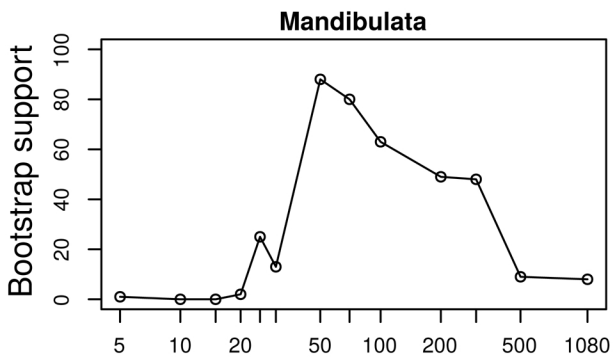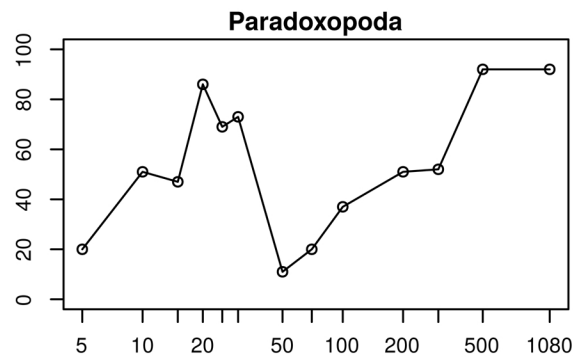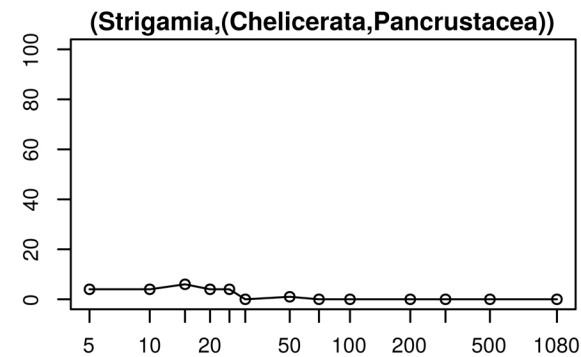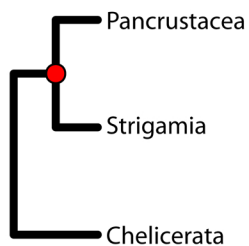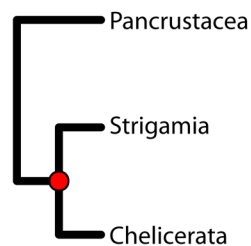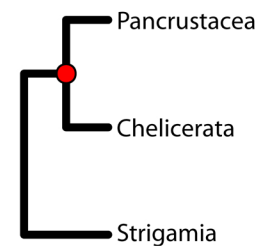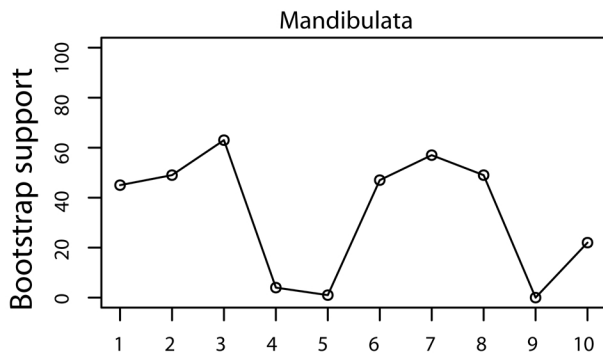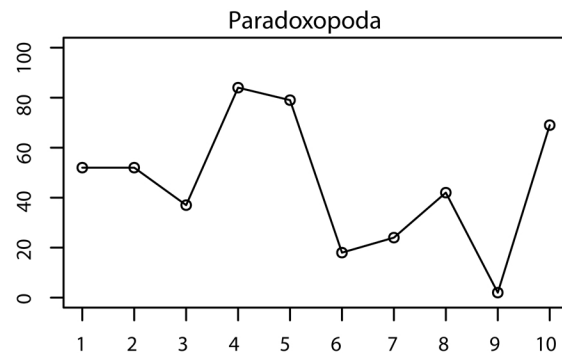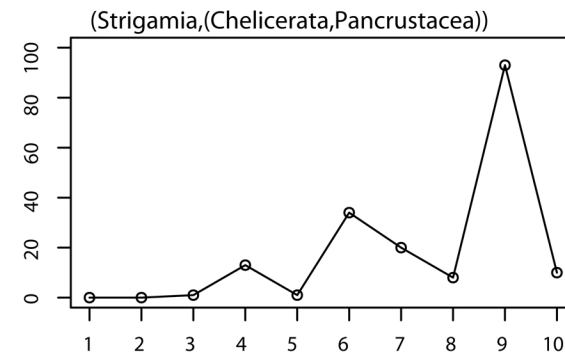

Supplement: Additional file 3: Figure S2. — Maximum Likelihood analyses of each type of filtered dataset. (PDF 2041 kb) [file 12864_2015_2146_MOESM3_ESM.pdf]

**A**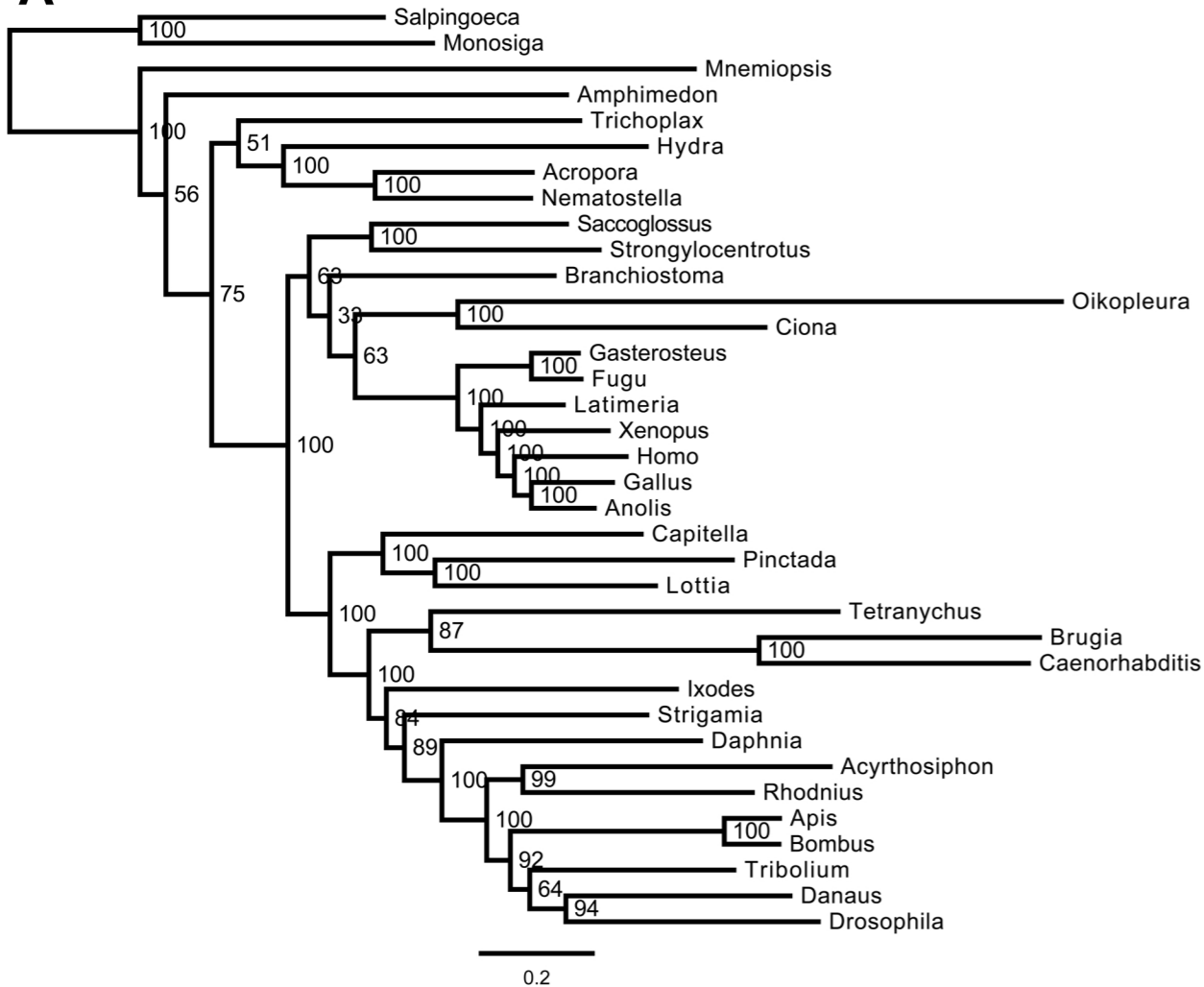**B**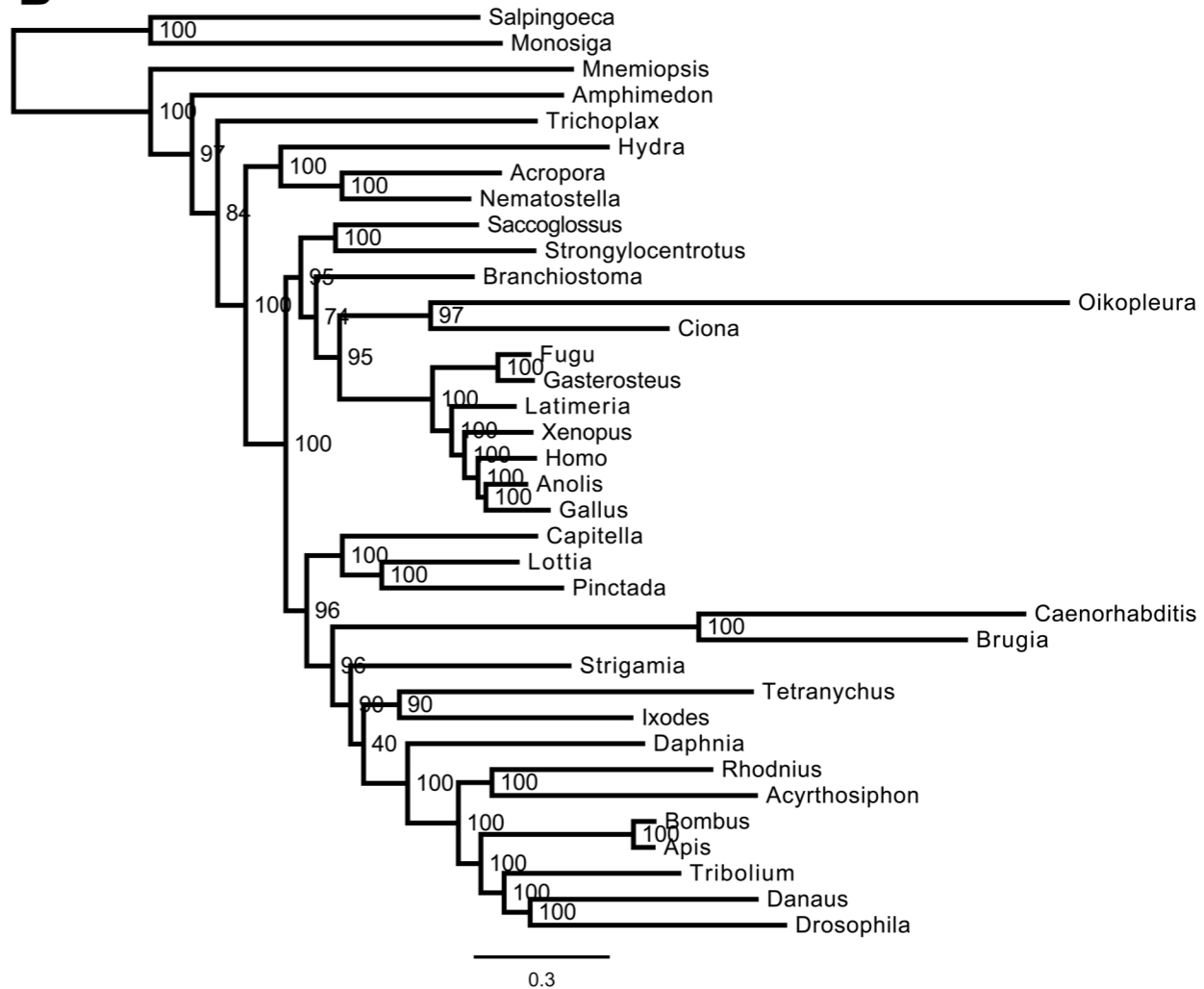

Supplement: Additional file 6: Figure S5. — Progressive concatenation and binning analysis of the position of Strigamia. (PDF 2880 kb) [file 12864_2015_2146_MOESM6_ESM.pdf]
