# Supplementary material for: Extracting phylogenetic signal and accounting for bias in whole-genome data sets supports the Ctenophora as sister to remaining Metazoa
Source: BMC Genomics. 2015 Nov 23;16:987. doi: 10.1186/s12864-015-2146-4 (PMC4657218; doi:10.1186/s12864-015-2146-4)
Supplement: Additional file 4: Figure S3. — Bayesian CAT-GTR trees of each type of filtered and recoded dataset. (PDF 7912 kb) [file 12864_2015_2146_MOESM4_ESM.pdf]

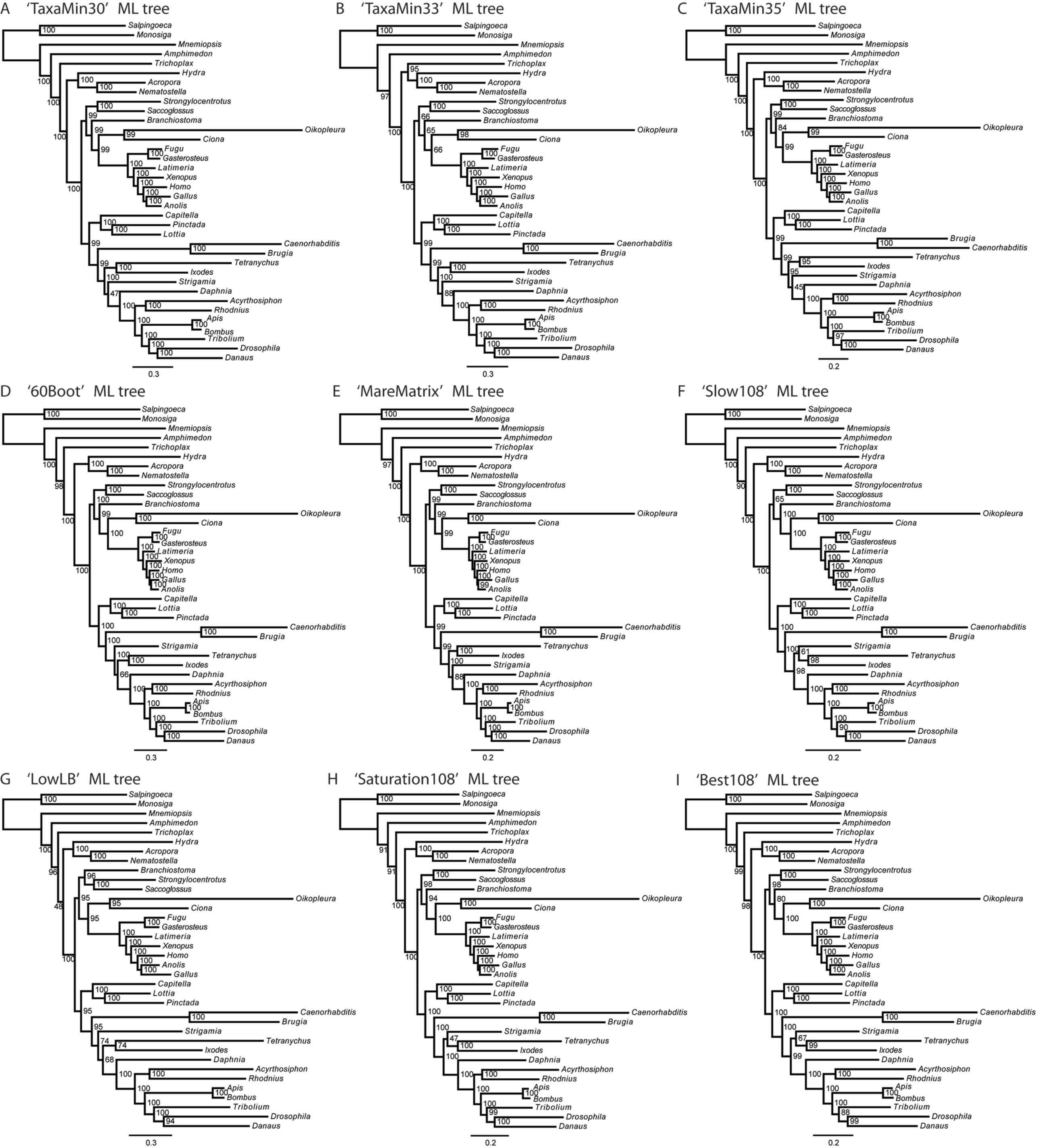

A 'TaxaMin30' ML tree
Salpingoeca Monosiga Mnemiopsis Amphimedon Trichoplax Hydra Acropora Nematostella Strongylocentrotus Saccoglossus Branchiostoma Oikopleura Ciona Fugu Gasterosteus Latimeria Xenopus Homo Gallus Anolis Capitella Pinctada Lottia Caenorhabditis Brugia Tetranychus Ixodes Strigamia Daphnia Acyrthosiphon Rhodnius Apis Bombus Tribolium Drosophila Danaus
0.3
B 'TaxaMin33' ML tree
Salpingoeca Monosiga Mnemiopsis Amphimedon Trichoplax Hydra Acropora Nematostella Strongylocentrotus Saccoglossus Branchiostoma Oikopleura Ciona Fugu Gasterosteus Latimeria Xenopus Homo Gallus Anolis Capitella Lottia Pinctada Caenorhabditis Brugia Tetranychus Ixodes Strigamia Daphnia Acyrthosiphon Rhodnius Apis Bombus Tribolium Drosophila Danaus
0.3
C 'TaxaMin35' ML tree
Salpingoeca Monosiga Mnemiopsis Amphimedon Trichoplax Hydra Acropora Nematostella Strongylocentrotus Saccoglossus Branchiostoma Oikopleura Ciona Fugu Gasterosteus Latimeria Xenopus Homo Gallus Anolis Capitella Lottia Pinctada Brugia Caenorhabditis Tetranychus Ixodes Strigamia Daphnia Acyrthosiphon Rhodnius Apis Bombus Tribolium Drosophila Danaus
0.2
D '60Boot' ML tree
Salpingoeca Monosiga Mnemiopsis Amphimedon Trichoplax Hydra Acropora Nematostella Strongylocentrotus Saccoglossus Branchiostoma Oikopleura Ciona Fugu Gasterosteus Latimeria Xenopus Homo Gallus Anolis Capitella Lottia Pinctada Caenorhabditis Brugia Strigamia Tetranychus Ixodes Daphnia Acyrthosiphon Rhodnius Apis Bombus Tribolium Drosophila Danaus
0.3
E 'MareMatrix' ML tree
Salpingoeca Monosiga Mnemiopsis Amphimedon Trichoplax Hydra Acropora Nematostella Strongylocentrotus Saccoglossus Branchiostoma Oikopleura Ciona Fugu Gasterosteus Latimeria Xenopus Homo Gallus Anolis Capitella Lottia Pinctada Caenorhabditis Brugia Tetranychus Ixodes Strigamia Daphnia Acyrthosiphon Rhodnius Apis Bombus Tribolium Drosophila Danaus
0.2
F 'Slow108' ML tree
Salpingoeca Monosiga Mnemiopsis Amphimedon Trichoplax Hydra Acropora Nematostella Strongylocentrotus Saccoglossus Branchiostoma Oikopleura Ciona Fugu Gasterosteus Latimeria Xenopus Homo Gallus Anolis Capitella Lottia Pinctada Caenorhabditis Brugia Strigamia Tetranychus Ixodes Daphnia Acyrthosiphon Rhodnius Apis Bombus Tribolium Drosophila Danaus
0.2
G 'LowLB' ML tree
Salpingoeca Monosiga Mnemiopsis Amphimedon Trichoplax Hydra Acropora Nematostella Branchiostoma Strongylocentrotus Saccoglossus Oikopleura Ciona Fugu Gasterosteus Latimeria Xenopus Homo Anolis Gallus Capitella Lottia Pinctada Caenorhabditis Brugia Strigamia Tetranychus Ixodes Daphnia Acyrthosiphon Rhodnius Apis Bombus Tribolium Drosophila Danaus
0.3
H 'Saturation108' ML tree
Salpingoeca Monosiga Mnemiopsis Amphimedon Trichoplax Hydra Acropora Nematostella Strongylocentrotus Saccoglossus Branchiostoma Oikopleura Ciona Fugu Gasterosteus Latimeria Xenopus Homo Gallus Anolis Capitella Lottia Pinctada Caenorhabditis Brugia Strigamia Tetranychus Ixodes Daphnia Acyrthosiphon Rhodnius Apis Bombus Tribolium Drosophila Danaus
0.2
I 'Best108' ML tree
Salpingoeca Monosiga Mnemiopsis Amphimedon Trichoplax Hydra Acropora Nematostella Strongylocentrotus Saccoglossus Branchiostoma Oikopleura Ciona Fugu Gasterosteus Latimeria Xenopus Homo Gallus Anolis Capitella Lottia Pinctada Caenorhabditis Brugia Strigamia Tetranychus Ixodes Daphnia Acyrthosiphon Rhodnius Apis Bombus Tribolium Drosophila Danaus
0.2
