# Supplementary material for: Extracting phylogenetic signal and accounting for bias in whole-genome data sets supports the Ctenophora as sister to remaining Metazoa
Source: BMC Genomics. 2015 Nov 23;16:987. doi: 10.1186/s12864-015-2146-4 (PMC4657218; doi:10.1186/s12864-015-2146-4)
Supplement: Additional file 5: Figure S4. — Comparison between trees derived from loci with mitochondrial (A) or nuclear (B) expression (cellular components). (PDF 10799 kb) [file 12864_2015_2146_MOESM5_ESM.pdf]

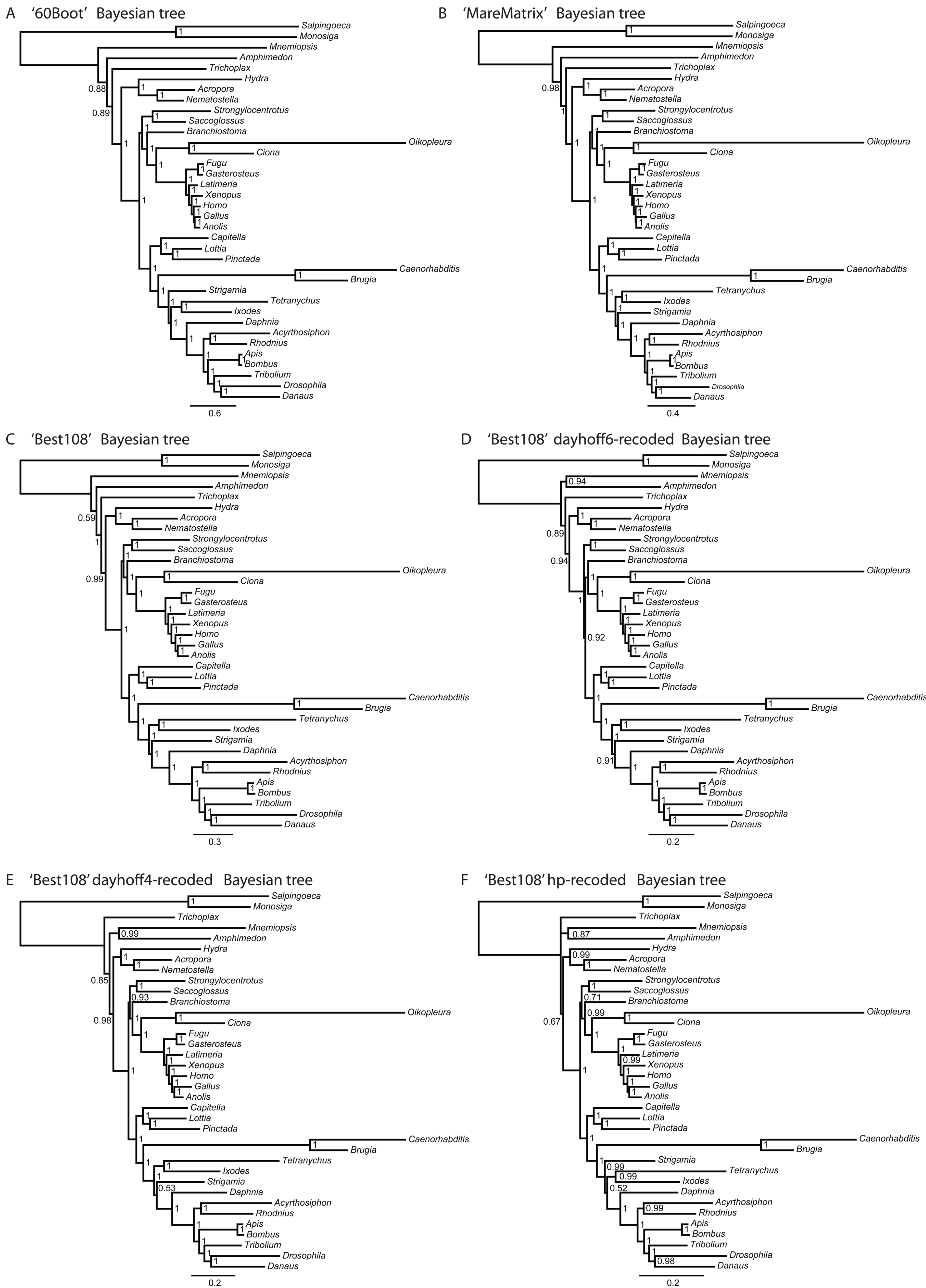
A ‘60Boot’ Bayesian tree
Salpingoeca
Monosiga
Mnemiopsis
Amphimedon
Trichoplax
Hydra
Acropora
Nematostella
Strongylocentrotus
Saccoglossus
Branchiostoma
Oikopleura
Ciona
Fugu
Gasterosteus
Latimeria
Xenopus
Homo
Gallus
Anolis
Capitella
Lottia
Pinctada
Caenorhabditis
Brugia
Strigamia
Tetranychus
Ixodes
Daphnia
Acyrthosiphon
Rhodnius
Apis
Bombus
Tribolium
Drosophila
Danaus
0.6
B ‘MareMatrix’ Bayesian tree
0.4
C ‘Best108’ Bayesian tree
0.3
D ‘Best108’ dayhoff6-recoded Bayesian tree
0.2
E ‘Best108’ dayhoff4-recoded Bayesian tree
0.2
F ‘Best108’ hp-recoded Bayesian tree
0.2
