# Supplementary material for: Extracting phylogenetic signal and accounting for bias in whole-genome data sets supports the Ctenophora as sister to remaining Metazoa
Source: BMC Genomics. 2015 Nov 23;16:987. doi: 10.1186/s12864-015-2146-4 (PMC4657218; doi:10.1186/s12864-015-2146-4)
Supplement: Additional file 7: Table S2. — Summary of statistics from Bayesian searches of phylogeny using PhyloBayes. Maxdiff < 0.1: good run, maxdiff < 0.3: acceptable. (PDF 11 kb) [file 12864_2015_2146_MOESM7_ESM.pdf]

Supplementary table 2. Details of the PhyloBayes analyses under CAT-GTR.

Matrix	Number of cycles (min. of 2 chains)	bpcomp maxdiff	burn-in
60boot	6501	0.0944444	2000
MareMatrix	5020	0.0324001	2000
Best108	8959	0.0759295	4000
Best108 hp-recoded	12886	0.0397183	1000
Best108 dayoff4-recoded	6500	0.0485092	1000
Best108 dayoff6-recoded	7994	0.0554071	1000

maxdiff < 0.1: good run, maxdiff < 0.3: acceptable.
