# Supplementary material for: Extracting phylogenetic signal and accounting for bias in whole-genome data sets supports the Ctenophora as sister to remaining Metazoa
Source: BMC Genomics. 2015 Nov 23;16:987. doi: 10.1186/s12864-015-2146-4 (PMC4657218; doi:10.1186/s12864-015-2146-4)
Supplement: Additional file 8: — Image attributions. (PDF 12 kb) [file 12864_2015_2146_MOESM8_ESM.pdf]

Figure 2 image attributions. All silhouettes in Figure 2 come from http://phylopic.org. Ctenophore image by Noah Schlottman. Sponge image by Mali'o Kodis, adapted from photograph by Derek Keats (http://www.flickr.com/photos/dkeats/). *Trichoplax* image by Michele Sites. Scyphozoan image by Mali'o Kodis, adapted from photograph by Ching (http://www.flickr.com/photos/36302473@N03/). Echinoderm image by Didier Descouens (vectorized by T. Michael Keesey). *Branchiostoma* (lancelet), hemichordate peanut worm images by Michele Site. Tunicate image by Mali'o Kodis adapted from a photograph by Melissa Frey. Nematode image by Michelle Sites. Centipede image by Noah Schlottman, adapted from photo by Carol Cummings. Heteropteran bug image by Joseph Hughes. Butterfly image by Fritz Geller-Grimm (vectorized by T. Michael Keesey). The credited images are available under the Creative Commons Attribution-NonCommercial 3.0 Unported, Creative Commons Attribution 3.0 Unported, or Creative Commons Attribution-NonCommercial-ShareAlike 3.0 Unported licenses [links: http://creativecommons.org/licenses/by-nc/3.0/, http://creativecommons.org/licenses/by/3.0/, http://creativecommons.org/licenses/by-nc-sa/3.0/]. All other images used have no copyright.
